# Treatment Techniques to Reduce Cardiac Irradiation for Breast Cancer Patients Treated with Breast-Conserving Surgery and Radiation Therapy: A Review

**DOI:** 10.3389/fonc.2014.00327

**Published:** 2014-11-14

**Authors:** Robert E. Beck, Leonard Kim, Ning J. Yue, Bruce G. Haffty, Atif J. Khan, Sharad Goyal

**Affiliations:** ^1^Department of Radiation Oncology, Rutgers Robert Wood Johnson Medical School, Rutgers Cancer Institute of New Jersey, New Brunswick, NJ, USA

**Keywords:** breast cancer, radiation, heart, dosimetry, cardiotoxicity

## Abstract

Thousands of women diagnosed with breast cancer each year receive breast-conserving surgery followed by adjuvant radiation therapy. For women with left-sided breast cancer, there is risk of potential cardiotoxicity from the radiation therapy. As data have become available to quantify the risk of cardiotoxicity from radiation, strategies have also developed to reduce the dose of radiation to the heart without compromising radiation dose to the breast. Several broad categories of techniques to reduce cardiac radiation doses include breath hold techniques, prone positioning, intensity-modulated radiation therapy, and accelerated partial breast irradiation, as well as many small techniques to improve traditional three-dimensional conformal radiation therapy. This review summarizes the published scientific literature on the various techniques to decrease cardiac irradiation in women treated to the left breast for breast cancer after breast-conserving surgery.

## Introduction

The American Cancer Society estimates that in 2014 about 232,000 new cases of invasive breast cancer will be diagnosed, as well as 62,500 cases of breast carcinoma *in situ* (1). The majority of these women will receive breast-conserving surgery followed by radiation. Breast irradiation has been shown to decrease the risk of local recurrence after breast-conserving surgery with few adverse effects (2). One of the most concerning complications of breast radiotherapy is cardiotoxicity from radiation to the heart.

Early studies showed decreased left ventricular function in breast cancer patients treated with radiation (3). Excess risk of cardiac mortality due to radiation, from two European randomized trials involving five different techniques, has been estimated to be 1.8% (4), though this data also suggested that only heart doses greater than 30 Gray (Gy) were important to calculate risk of cardiac toxicity. Cardiotoxicity is most frequently reported as decreased myocardial function or coronary artery disease (also reported as ischemic heart disease or decreased cardiac perfusion). However, less common toxicities can include myocardial infarction, congestive heart failure, pericarditis, arrhythmias, angina, or valve dysfunction (5, 6). While generalized decreased cardiac function has been generally reported, some studies in this review have specifically shown decreased left ventricular or left anterior descending coronary artery (LAD) function or perfusion after radiation.

A review of over 1600 patients with 16 years of follow-up found that left-sided breast cancer patients treated with radiation had a 38% increase in cardiovascular disease compared to right-sided cancer patients, though the rates of cardiovascular disease did not correlate with volume of heart irradiated (7). Recently, another review of 2168 women who underwent radiotherapy for breast cancer in Sweden and Denmark found that the average mean heart dose was 4.9 Gy and that there was a significant linear correlation between mean heart dose and rate of major coronary events, with an increase of 7.4% per Gy (8). Another study estimated the risk of cardiotoxicity to increase 4% per Gy mean heart dose (9).

It should be remembered that for patients with long follow-up, the treatment techniques used may be relatively outdated compared to those used today, and therefore, their reported cardiac doses may not represent typical doses today. In addition, for such patients, 3D dose and image data, which are routinely available today, were generally not available in many older studies, requiring more uncertain methods of estimating cardiac dose. While rates of cardiotoxicity are improving, and methods of delivering and quantifying dose of radiation to the heart have become more sophisticated, reducing potential for any cardiotoxicity remains one of the primary aims of improving adjuvant radiation techniques for patients with left-sided cancers.

This paper will focus on treatment of patients treated with radiation after breast-conserving surgery. Treatment fields, angles, and other radiotherapy techniques may be different for post-mastectomy patients compared to patients with intact breasts. It is beyond the scope of this paper to attempt to discuss all aspects of plan evaluation for the studies discussed, such as planning target volume coverage, dose homogeneity, and dose to other organs. This review will focus solely on techniques to decrease radiation to the heart for women receiving radiation to the left breast.

## Materials and Methods

A Pubmed literature search was performed on March 5, 2014 to review any papers discussing breast cancer heart dosimetry. Articles were excluded if they reviewed non-breast cancer data, post-mastectomy radiation, exclusively evaluated patients with pectus excavatum, bilateral breast irradiation, or did not have heart dosimetric data. Articles were reviewed specifically for data from patients treated to the left breast. For this review, all studies are assumed to deliver whole breast irradiation unless partial breast treatment is stated.

## Results

### Supine 3D

Traditionally, breast cancer has been treated in the supine position with arms above the head with two opposed tangent photon fields. The earliest data on cardiac toxicity originated from the Stockholm Breast Cancer trial, which treated patients to 45 Gy at 1.8 Gy per fraction, and found a 15-year excess cardiac mortality of 6.8% attributed to the radiation (10). A review of patients treated in that trial estimated the mean volume of heart treated to the 50% isodose (22.5 Gy) to be 25% (11). One of the first trials to show an alternative approach to reduce heart dose was a review of the plans of 100 women with left-sided T1N0MO breast cancer status post-lumpectomy treated with three-dimensional conformal radiation therapy (3DCRT) planning to 50 Gy at 2 Gy per fraction, which reviewed the dose to the heart for these patients and found the volume treated to 50% isodose to be 5.7% (approximately 33 cc) (12). This significant reduction of heart dose led to the widespread adoption of 3D conformal planning for breast cancer. Some have shown that simply using 3DCRT to account for individual organ location, by putting a limit of 1 cm of heart in the tangent field, would cause at most a 1 per thousand patient risk of cardiac mortality (13). Several other studies have also shown reductions in planned heart dose with 3D conformal compared to two-dimensional planning (14, 15). However, one study showed no difference in mean heart dose, V20, or V5 heart dose comparing 2D, standard 3DCRT, and field-in-field (FiF) techniques (16).

Since the adoption of 3DCRT, many techniques have been attempted to further reduce cardiac radiation dose. A large study involving 217 left-sided breast cancer patients evaluated 3DCRT vs multi-segmented conformal radiation therapy and found no difference in mean heart dose (17). Another study confirmed this finding (18). A study evaluating tangential single wedge, double wedge, and FiF techniques found no significant differences in cardiac dose (19). A single study evaluating treating women with large breasts in the left lateral decubitus position was able to achieve a mean heart dose of 1.35 Gy for left-sided cancers (20). Using FiF planning can produce lower heart mean dose, V10, and V20 compared to standard 3DCRT plans (21). One study found that treating patients with their bra on decreased V5 to the heart from 9.8 to 2.7% (22). Hypofractionated whole breast regimens are becoming more common and have been shown to have equal slightly improved 2 Gy dose equivalent doses to the heart (23, 24).

### Prone

The largest and most current experience with prone breast treatment includes 200 women with left-sided breast cancer and has shown a significant decrease in in-field heart volumes compared to supine tangent plans with a mean reduction of 7.5 cm^3^, which corresponded to a 85.7% reduction in in-field heart volume (25). However, there was no benefit for women with smaller breasts (less than 750 cm^3^), and 15% of women overall had decrease in in-field heart volume when planned in the supine position. The second largest study comparing supine and prone planning, comparing whole breast and partial breast plans, found that prone positioning decreased cardiac doses for large breasted women but increased cardiac doses for women with smaller breast volume (26), a finding that has also been concluded in other studies (27, 28). One study found improvement in heart doses with prone positioning, but at the cost of a 50% reduction in coverage of the axillary nodes (29). Some smaller series have found no difference between supine and prone heart doses (29–31). Figure 2 provides examples of prone breast and an external beam accelerated partial breast irradiation (APBI) plans with corresponding isodose lines.

### Intensity-modulated radiation therapy

As has been shown in many sites treated with intensity-modulated radiation therapy (IMRT), left-sided breast cancer patients treated with IMRT limits high dose to the heart without limiting low doses (32–35). Different techniques, including forward-planned IMRT, inverse-planned IMRT, and modulated arc therapies have been studied. A study of multiple partial arc volume-modulated arc therapy had a mean V25 to the heart of 2.52% of the heart volume, while having a mean total dose of 7.61 Gy (36). IMRT incorporating a simultaneous boost, even with respiratory gating, showed a mean heart dose of 22.98 Gy but reduced treatment duration by 6 fractions (37). Whether standard sequential boost or IMRT concomitant boost was used did not significantly affect heart dose (38). Forward-planned IMRT has been shown in one study to significantly reduce mean heart dose compared to inverse IMRT and arc radiotherapy (5.46 vs 15.48 vs 12.73 Gy) (39).

Many studies comparing IMRT to 3DCRT have shown decreased heart mean, V25, and V30 with IMRT compared to standard tangent fields (40–47), however, with no improvement over tangents with FiF (48). Other studies have failed to show a significant difference in most heart constraints for IMRT over 3DCRT (49). The largest study comparing 3DCRT vs IMRT, comparing 201 forward-planned IMRT cases to 131 3DCRT plans, stratified by breast size and use of supraclavicular nodal irradiation, found a non-significant trend toward reduced heart constraints with IMRT (50).

### Technological solutions

**Figure 1:**
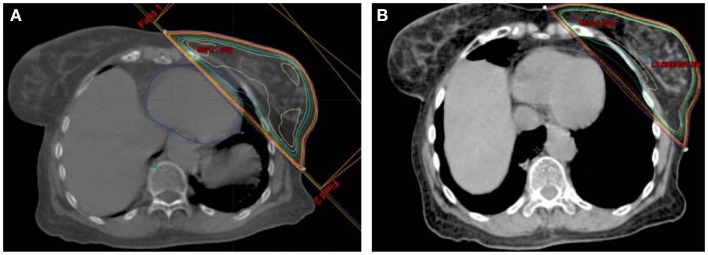
**Example of (A) free breathing and (B) deep inspiration breath hold plans for a single patient**.

Breath hold, accomplished by having the patient take and hold a deep inspiration during CT simulation and during treatment each day, has been shown to significantly reduce heart dose. Several studies have shown that deep inspiration breath hold (DIBH) compared to free breathing (FB) reduced mean heart dose and several other dose constraints to the heart by 50%, with mean heart doses around 2–3 Gy (51–55). A comparison of thoracic anatomy and radiation isodose lines with FB and DIBH can be seen in Figure 1, which demonstrates how the breath hold can change thoracic anatomy to potentially reduce cardiac dose received of radiation. A selective approach to using DIBH was used in one study, which evaluated 53 left breast patients and evaluated all patients with standard tangent field plans. Any patients with greater than 10 cm^3^ of heart receiving 50% of the prescription dose were selected for DIBH IMRT, and these DIBH IMRT cases had significantly reduced whole heart and LAD doses (56). One study combined DIBH with IMRT and significantly reduced heart V30 in two-thirds of the patients and was able to avoid any heart irradiation in 22% of cases (57). Another study, using cardiac MRI, similarly found that breath hold could displace the heart entirely out of the radiation field in 21% of patients (58).

**Figure 2:**
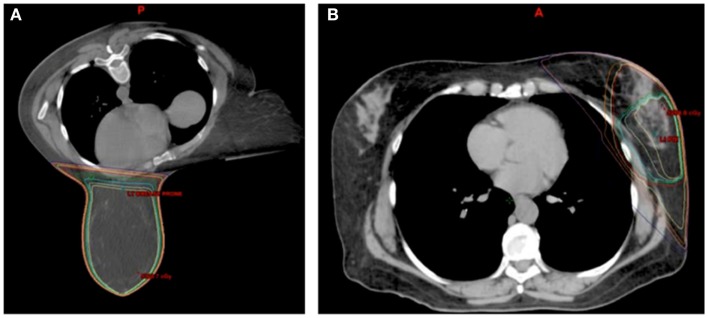
**Examples of (A) prone breast and (B) external beam APBI plans**.

One consideration of breath hold techniques is inter-fraction reproducibility of patient geometry and anatomy. When the breath hold is voluntary, respiratory coaching is required to ensure consistency. Two studies have shown good inter-fraction reproducibility with DIBH (53, 59). Monitoring technology such as magnetic sensors or real-time surface imaging can be used to verify and improve voluntary breath hold reproducibility (59, 60). Several studies rely on technology sometimes referred to as active breathing control, in which a patient breathes through a device that monitors breathing air volumes and automatically holds the patient’s breath at pre-specified volumes for a defined period of time (55, 57, 61–64). Some studies have explored the use of gating rather than breath hold to address intra-fraction respiratory motion (60, 65). Even with respiratory motion management such as breath hold, cardiac motion may still be an issue. Under breath hold conditions, one study showed that the LAD can show substantial displacement due to cardiac contraction (66). Another study used fluoroscopy to show potentially significant cardiac motion that was not evident using 4DCT techniques (67).

Changing the radiation particle from photons to protons and using MRI-linacs for photon treatment delivery are two newer approaches to improving treatment delivery. Proton radiotherapy is not commonly used for the breast; however, one study projected that a reduced risk of cardiac mortality might be achieved, based on planned cardiac doses, for proton and IMRT plans compared to 3DCRT (68). A study of breast radiotherapy using integrated MRI-linacs found no difference in heart D2cc or V25 for whole breast tangential and 7-field IMRT APBI plans (69). One potential application for future MRI-linacs is the appropriate application of a reversible transverse magnetic field, which in simulation resulted in a 26.0% mean heart dose reduction (70).

### APBI

Accelerated partial breast irradiation is a newer technique in women with low risk of recurrence for breast cancer to treat only the lumpectomy cavity with a small margin, rather than the whole breast and regional lymph nodes. Only women at least 60 years old with T1, node negative, estrogen receptor positive, unifocal or unicentric breast cancers with no lymphovascular invasion and negative margins are fully “suitable” for APBI, with a select group also considered “cautionary,” per the American Society for Radiation Oncology (71). More recently, APBI guidelines were also created by the American Brachytherapy Society with slightly different criteria for “suitable” patients, such as slightly older age and no DCIS allowed (72). However, these guidelines are created by consensus panels for patients off protocol, rather than by randomized trials with set selection criteria. While APBI is only available for a select group of breast cancer patients, it is often able to significantly reduce dose to nearby structures including the heart.

A few studies have compared whole breast irradiation to ABPI. A study evaluating APBI using IMRT compared to whole breast using FiF planning (using radiobiologically adjusted results to account for the different fractionations) found that the APBI plan reduced the heart mean from 3.17 to 0.80 Gy (*p* = 0.002) using APBI and reduced V5 from 8.75 to 4.94% (*p* = 0.041) (73). Another review of patients being treated on NSABP-B39 for external beam APBI compared to plans for whole breast irradiation has significantly improved V2.5, V5, and V10 for lateral lumpectomy cavities but not for medial cavities, though V20 was improved with APBI regardless of lumpectomy location (74). Mammosite brachytherapy APBI compared to whole breast irradiation has been shown to significantly reduce maximum heart dose and V5, but not mean heart dose or V10 in one study (75), but single-source APBI brachytherapy did show an improvement in mean heart dose over whole breast from 2.52 to 1.65 Gy in another study (76).

Accelerated partial breast irradiation can be delivered via external beam radiation or via brachytherapy catheter(s) placed in the lumpectomy cavity. Studies of brachytherapy APBI have shown mean heart dose between 1.65 and 2.45 Gy and mean V5 between 1 and 59.2% (77–80). One study achieved a mean maximum heart doses around 2.2 Gy in both Mammosite and Clearpath brachytherapy catheters, though patients in this study have lesions closer to skin than chest wall (81). External beam studies have shown mean heart doses of 1.2 Gy and V5 of 1% (82, 83). RTOG 0413 showed external partial breast irradiation with a mean V5 value at 1.1% for left-sided patients (84).

Accelerated partial breast irradiation with protons has been shown to be very effective at limiting heart dose with one study showing no dose greater than 3 Gy to the heart (85). One study evaluating volumetric-modulated arc radiotherapy (VMAT) was able to achieve an APBI plan with a mean heart dose of 0.72 Gy, which was further reduced to 0.34 Gy (a 53% reduction) when VMAT was combined with dynamic couch rotation to account for respiratory motion (86). When evaluating IMRT, VMAT, and continuous arc rotation of the couch APBI plans separately, compared to a 3DCRT APBI plan, the IMRT and continuous arc plans were able to significantly reduce the mean heart V5 from 3 to 1.1% and 1.7%, respectively (87). Another study found that for pendulous breasts treated prone with IMRT APBI combined with dynamic couch motion could produce a plan that would deliver less than 0.1% of the prescribed dose to the heart (88).

### Internal mammary node and boost cardiac contribution

Slight variations in dose exist though most studies are close to a biologic equivalent dose (at 2 Gy per fraction) of 50 Gy, though a significant variation in the implementation and dose of a boost to the surgical bed exist between studies. Another difficulty in evaluating cardiac dose is variability in treatment volume. Variability in coverage of internal mammary nodes (IMN), axillary, or supraclavicular nodes exists between studies. Adding axillary nodal or IMN coverage to tangent fields has been shown to increase the Dmax of the heart by 7–10% (89). Adding IMN coverage to whole breast irradiation increases the volume of heart irradiated by 13.8% for left breast cancers (90). When comparing plans with IMN in the treatment field, one study found no difference between wide-field, oblique photon-electron, and perpendicular photon-electron techniques (91), while another found decreased mean heart dose, V10, and V20 with wide tangents compared to plans using a separate IMN field (92).

The use and dose of a boost to the lumpectomy cavity is not standardized between studies, nor among practitioners, which contributes to the difficulty in comparing studies. The added mean heart dose of a 10 Gy boost in four fractions is 0.33 Gy for electron boost and 0.73 Gy for a photon boost via VMAT (93). Other studies have found decreased cardiac doses for proton and photon compared to electron boosts (94), and comparable V20 for Mammosite brachytherapy boost compared to electron boost (95).

## Discussion

One of the difficulties in comparing studies in radiation cardiac toxicity is the variable reported parameters to evaluate potential toxicity. For example, as mentioned previously, early studies evaluated the volume receiving 50% of the prescription dose (10–12). Another study reviewed SPECT perfusion scans in 20 women 6 months after breast radiation and found minimal decrease in perfusion if RT dose was kept less than 10 Gy and a 20% perfusion reduction if greater than 40 Gy (96), suggesting V10 and V40 as potential targets for plan evaluation. Strain rate imaging has also been used to evaluate cardiac damage from radiation and has shown that radiation of left breast patients led to a significant 2% reduction in left ventricle strain after radiation, particularly observed in regions of the heart exposed to 3 Gy or more (97), which was observed immediately after radiation and persistent when evaluated 14 months after radiation (98). Cardiac biomarkers have also been evaluated following breast irradiation, and while there was a significant increase in mean values of troponin I and Brain Natriuretic Peptide (from 0.007 to 0.014 ng/mL and 123 to 159 pg/mL, respectively), the increase was not above normal reference values (99). A study of 681 breast cancer patients treated in Denmark who did not develop ischemic heart disease found that left-sided breast cancer patients had a mean heart dose of 6 Gy, despite receiving coverage of IMN and supraclavicular fields (100). Mean heart dose is also used as a common reference dose constraint given reports of clinical outcomes in studies using this parameter (8). Table 1 provides a comparison of many studies that included mean heart dose data; however, caution should be used in comparing studies, as many studies included low numbers of patients, and extent of breast and nodal tissue covered differs from one study to another. Studies have failed to consistently show that LAD dose is independently predictive of cardiotoxicity more than whole heart measures and more reproducible from one physician to another. Therefore, whole heart dose remains a standard measure at present. However, further data are needed to more rigorously establish standards for dosimetric cardiac constraints.

**Table 1:** **Summary of studies evaluating mean heart dose**.

Reference	*n*	Treatment technique	Mean heart dose (Gy)
(19)	15	3DCRT (with 16 Gy boost). Tangential single wedge vs double wedge vs FiF	3.31 vs 3.31 vs 3.07
(16)	15	2D vs 3D vs FiF	4.42 vs 5.33 vs 5.17
(101, 102)	358	3DCRT	5.1 if treated in 1950s and 3.0 Gy if treated in 1990s
(17)	217	3DCRT vs multi-segmented conformal radiation therapy	4.8 vs 4.8
(103)	50	3DCRT	2.3
(92)	32	3DCRT including IMNs: 2 plans with separate IMN fields vs wide tangents	6.4 vs 8.1 vs 3.8
(21)	10	Bilateral wedge tangents vs FiF	2.2 vs 1.89
(20)	26	3DCRT in left lateral decubitus position	1.35
(55)	87	3DCRT vs moderate DIBH using active breathing control	4.23 vs. 2.54
(56, 66)	53	3DCRT, if V50 > 10 cm^3^, then DIBH IMRT	3.17 vs 1.32
(51)	30	IMRT with simultaneous integrated boost in free breathing and DIBH	6.9 vs 3.9
(52)	12	FB vs DIBH	6.2 vs 3.1
(28)	12	Prone vs supine: wedged tangents, FiF, and multibeam IMRT	Wedged tangents: 1.9 vs 3.9. FiF: 1.6 vs 3.3. IMRT: 1.6 vs 2.5
(104)	5	Prone tomotherapy IMRT	8.7
(36)	10	Multiple partial volumetric-modulated arc therapy technique	7.61
(37)	24	Respiratory gated simultaneous integrated boost IMRT	22.98
(39)	10	Forward-IMRT vs inverse-IMRT vs intensity-modulated arc radiotherapy	5.46 vs 15.48 vs 12.73
(33)	20	Small breasted women treated with wedged tangents vs FIF vs T-IMRT vs M-IMRT vs VMAT	3.7 vs 3.2 vs 2.2 vs 4.4 vs 4.6
(48)	10	3DCRT vs tomotherapy IMRT vs FiF	4.0 vs 3.0 vs 3.0
(38)	11	Hypofractionated concomitant boost radiotherapy using IMRT vs standard sequential boost technique	2.2 vs 3.2
(42)	13	Tomotherapy vs 3DCRT	1.35 vs 2.22
(44)	14	3D vs IMRT for unfavorable thoracic geometry patients	6.85 vs 8.52
(73)	12	APBI IMRT vs 3DCRT with FiF	0.80 vs 3.17
(75)	6	Mammosite HDR brachytherapy APBI vs 3DCRT	3.5 vs 3.8
(76)	26	Single-source HDR brachytherapy APBI vs 3DCRT	2.52 vs 1.65
(80)	60	Brachytherapy ABPI	2.45
(82)	25	External beam APBI (2 minitangent beams and en face electron beam)	1.2
(93)	14	Dose contribution from 10 Gy/4 fraction boost using few leaf electron collimator-based modulated electron radiotherapy vs conventional direct electron vs VMAT	0.34 vs 0.33 vs 0.73

**n*: number of left-breast women in study (total number if left breast not specified); 3DCRT: three-dimensional conformal radiation therapy; FiF: field-in-field; IMN: internal mammary nodes; DIBH: deep inspiration breath hold; IMRT: intensity-modulated radiation therapy; FB: free breathing; APBI: accelerated partial breast irradiation; FB: free breathing; HDR: high dose rate; VMAT: volumetric-modulated arc therapy*.

Another challenge is defining the volume used to calculate these dose constraints. Slight variations in heart contours can exist from one radiation oncologist to another. Also, some have questioned whether it may be valuable to contour the LAD or other coronary vessels individually and whether to include the pericardium. For this reason, a heart atlas for CT contouring, developed jointly by cardiology, cardiac radiology, and radiation oncology, to delineate whole heart and separate coronary vessels, has been shown to improve accuracy of cardiac contours, and more consistent mean heart dose reporting, in a tested group of radiation oncologists (105). While this atlas was verified in a group, it is not used by all radiation oncologists and has not been used for contouring in other studies evaluating heart data, because such atlases are still relatively new. User contour variations, therefore, exist between studies. Some studies have suggested that the maximum heart distance in a treatment field, measured anterior to posterior, is relatively simple and correlates well with mean heart dose and other cardiac dose measurements (101, 106). However, another study showed that maximum heart distance only correlated with dose to the LAD when accounting for respiratory motion (107). A study of left-sided breast cancer patients where all plans had LAD, right, and circumflex coronary arteries contoured separately found that the mean whole heart dose was 2.3 Gy, and 7.6 Gy to the LAD, and 2 Gy to the right and circumflex arteries (103). A recent study of supine standard tangential field plans found that for every 100 cGy increase in mean heart dose the mean LAD dose increased by 4.82 Gy, with direct correlations also seen with several other constraints, suggesting that LAD dose correlates very closely with whole heart parameters and LAD does not need to be contoured separately (108). However, another study using 3 field mono-isocentric partial wide tangents found that 11 of 24 patients had significant variability between mean heart dose and LAD dose (109). A study of 32 women on a randomized trial, treated with breast radiotherapy, evaluated the cardiac perfusion before and 1 year after radiation, and found no significant change in cardiac perfusion after radiation, even when assessing various cardiac subvolumes (110).

Variability in dose planned to dose received can exist. It can be difficult to determine the actual dose received. However, some studies provide insight into means of limiting the variability between these doses. It has been shown that patient setup errors of greater than 3 mm in the posterior direction result in significant increased dose to the heart (111, 112). The maximum anterior/posterior distance of heart in the treatment field has shown a strong linear correlation with mean heart dose (100). Even with image guidance, planning margins may be advisable as variability can exist between bone and/or surface anatomy and cardiac (25, 60, 111).

The implementation of improving techniques for breast cancer radiotherapy can significantly reduce the heart radiation dose that breast cancer patients receive. A review of 358 women treated over several decades in Sweden found that even though a number of different treatment techniques were used, the overall mean heart dose to left-sided breast cancer patients was 5.1 Gy in the 1950s compared to 3.0 Gy for women treated in the 1990s (102). However, it should be remembered that even clear dosimetric advantages in the treatment planning stage may not translate to improvements in clinical outcomes (63).

Radiation is not the only factor contributing to cardiac toxicity in breast cancer patients, as other aspects of their treatment can influence cardiac toxicity. For example, a large study involving doxorubicin and cyclophosphamide chemotherapy with radiation to either the right or left breast (with or without IMN coverage) found that the number of cycles of doxorubicin was a more significant factor in cardiac toxicity than the amount of heart in the radiation field (113). Therefore, all aspects of patient care must be accounted for to reduce cardiac toxicity.

The decision of which treatment planning technique for delivery of radiotherapy following breast-conserving surgery includes consideration of many factors about the patient. One important factor in that decision is radiation doses received to the heart, as decreasing radiation doses to the heart can potentially prevent unnecessary cardiotoxicity. Many different techniques are available, as discussed in this review, to significantly reduce radiation doses to the heart, thereby providing means to decrease cardiac toxicity risk for women undergoing such treatment.

Several techniques have been shown to improve cardiac doses over standard supine 3DCRT tangents. Prone positioning has been shown to improve cardiac doses for patients with large pendulous breaths, though not for smaller breasted patients. Breath hold can also significantly reduce heart dose by displacing the heart away from the chest wall. APBI can be effective in reducing cardiac radiation doses though this is dependent on the location of the tumor/lumpectomy cavity and is only suitable for a select portion of breast cancer patients. Use of seroma boost and IMN irradiation has been shown to increase cardiac dose, though the cardiac risk needs to be weighed against the risk of recurrence.

## Conflict of Interest Statement

The authors declare that the research was conducted in the absence of any commercial or financial relationships that could be construed as a potential conflict of interest.
